# Supplementary material for: Spatial regression models to assess variations of composite index for anthropometric failure across the administrative zones in Ethiopia
Source: PLoS One. 2024 Feb 28;19(2):e0282463. doi: 10.1371/journal.pone.0282463 (PMC10901317; doi:10.1371/journal.pone.0282463)
Supplement: Attachment — Submitted filename: Comments.pdf [file pone.0282463.s001.pdf]

Review Report

This paper aims to evaluate the existence of spatial dependence on the outcome of undernutrition and the respective covariates in Ethiopia across the administrative zones. Specifically, to:

determine and explore the overall and local spatial dimension of CIAF among the under-five children in Ethiopia, (2) identify the factors and their effects for the spatial disparities in the average CIAF among the under-five children (3) compare the spatial model's performance to the standardized regression model, and (4) address the spatial vulnerability of the community to child undernutrition at the zonal level using the EDHS dataset.. The manuscript is written in a structured style, and is easy to follow. However, the language should be checked, and some aspects of the paper need extra work or clarification.

Please find the following specific comments:

Methods/Methodology

1-Although the authors drew the models in figure 3, the explanation of how the models evolve is not very clear. A clearer explanation of the models would be helpful in understanding the results from each.

2-In table 1, the authors mentioned that yi (CIAF) is binary variable and later the number of CIAF cases observed in the zone i (i=1, 2, . . . , 72), denoted by yi , was assumed to follow a Poisson distribution. Please, provide a clear explanation about that approximation.

3-The authors mentioned that it is spatial data, what are the sample sizes of these variables in each region.

4- The notations seems not consistent through the paper. For example, SAC: spatial autocorrelation model and SAR: spatial autoregressive model. GNS: general nesting spatial model and in table 3, what is GNNS?

5-In the methodology, the authors mentioned that "The spatial effects model is summarized in Fig.3". In that figure, OLS is mentioned. Is it a spatial effects model? It will be "The spatial effects models as well as the OLS model……"

6-Which software was used for the analysis?

7-Various methods can be used to estimate spatial models, which one was used in this article?

Abstract

1- Line 2 "………but the spatial heterogeneity in these relationships across the administrative zones remains unstudied". I find that this statement should be rewritten since according to Fenta et al. (2021). Research entitled "Spatial data analysis of

malnutrition among children under five years in Ethiopia", spatial heterogeneity was used in the models.

2- Line 4 "……zones autocorrelation structure in the under-five children's undernutrition studies. This should be rewritten.
3- Line 6. "We obtained the zonal-level …….. data for the under-five children in Ethiopia from the Ethiopian Demographic and Health Survey (EDHS) dataset". It will be "We used the zonal-level …. data for the under-five children in Ethiopia from the Ethiopian Demographic and Health Survey (EDHS) dataset".
4- The spatial autocorrelation model (SAC) was the best fit based on the AIC and BIC criteria. I am not able to identify the BIC values in the tables. I don't think if those values were generated.
5- The Confidence intervals are not shown in table 3.
6- Is mothers' literacy rate the same with % women with illiteracy in table 3? If yes, use the same name. Also, the value in the table and which described are not the same. 0.605 vs 0.065
7- Is minimum temperature the same with mint? The value described is not correct -0.158 vs -0.133. What about the p value?
8- …." The significant covariates may provide insights for policymakers for considerations." It should be rewritten.

Results

1-It would be helpful to include a map of Ethiopia with the administrative divisions shown the generated latent spatial effects with a Moran's I.

2- Note of the table 2, …. "*, ** and ***= Moran's I are significant at 10, 5, and 1% level". It will be ""*, ** and ***= Moran's I are significant at 10%, 5%, and 1% level, respectively".

3- What is N in table 3?

4- Use the same number of decimal. For example, "…. in Ethiopia was 52%, with the mothers' illiteracy rate of 73%, and with 38.44% …".

Discussion

What is U5C?

The results are not well discussed.

Decision

The paper can be published upon addressing the above comments.
