# Supplementary material for: Spatial regression models to assess variations of composite index for anthropometric failure across the administrative zones in Ethiopia
Source: PLoS One. 2024 Feb 28;19(2):e0282463. doi: 10.1371/journal.pone.0282463 (PMC10901317; doi:10.1371/journal.pone.0282463)
Supplement: Attachment — Submitted filename: Comment.docx [file pone.0282463.s002.docx]

**Spatial regression models to assess variations of the composite index for anthropometric failure across the administrative zones in Ethiopia**

Summary: Overall, the manuscript is good for statistical analysis or method paper. The authors were trying to assess the variation of CIAF across the different Zones of Ethiopia. Using a spatial regression model is a new technique to assess the CIAF variation. But it is unclear how this study could help the policy makers/ clinicians/ others to take necessary measures to reduce malnutrition. The public health background is absent for this paper, as CIAF is for the policymakers but not for clinicians. Here are some comments from the authors which would help the paper to be in better shape for the readers and policymakers.

Abstract: I am a bit confused, about how the authors write the conclusion based on the results shown in the abstract. “Results from the SAC model suggested that the mothers’ literacy rate (0.065, pvalue0.001) ….” What is 0.065? mentioning this would be easy for a reader to understand the values. The background section needs to write the importance of this study, the authors mainly mentioned the gap! Is there any other public health implication for conducting this study? Have any role to prevent malnutrition?

The keyword should contain CIAF.

Introduction: In the 1^st^ paragraph, the last two lines need references. The aim of the study is, it is a kind of method paper. Better to have some public health impact in the introduction section. How This can help to reduce malnutrition/ policymakers to understand the result of this study for taking mitigation strategy for tackling malnutrition.

Study area: Need references along with the figure. Why only 2000-2016 data were used? Not the recent one? Up to 2020 or more?

Variables of the study: How Z scores were calculated? Better to mention in this section, as CIAF is the important one in this paper. For readers who are from a non-statistical background, I think the table is not sufficient, the author can elaborate on the covariates; how they were collected, how they were categorized, and how defined.

Methods: Authors wrote the methods section in detail, which is highly appreciated.

Result: It is clear cut and shown in a good manner fashion.

Discussion: After reading the discussion section, it seems incomplete to me. It’s ok that no other research did not use this type of data, but authors can compare their findings with the studies outside Ethiopia, or other studies which are globally acceptable for similar types of research.

“In the SAC model, which is considered to be the best, the signs of below minimum dietary diversity score, illiteracy rate, the proportion of low BMI index, and precipitation variables were positive and significantly different from zero.” -are those findings very common and why these were observed among Ethiopia? It seems the result section is a bit more elaborated in the discussion section. The discussion needs to be rewritten.

Is there any strength or limitation of this analysis/ study? Which might be mentioned at the end of the discussion section.

Conclusion: “The spatial models allow the development of models that will significantly assist decision-makers in the improvement of undernutrition.”- I have no idea how? It is not clear in the conclusion/discussion section. Policymakers/ decision makers are not super specialized with statistical methods always. For the policy implication, a method paper is not a good option according to my knowledge. The recommendation should be changed or rewritten.

Minor comments: Language can be better, sometimes it seems sentences are alone, not meaningful. There were some issues with spacing and font.
