# Supplementary material for: Spatial regression models to assess variations of composite index for anthropometric failure across the administrative zones in Ethiopia
Source: PLoS One. 2024 Feb 28;19(2):e0282463. doi: 10.1371/journal.pone.0282463 (PMC10901317; doi:10.1371/journal.pone.0282463)
Supplement: Attachment — Submitted filename: Response to Reviewers.docx [file pone.0282463.s003.docx]

**RESPONSE THE REVIEWERS’ COMMENTS**

Dear Dr. Xiaohong Li
Academic Editor
PLOS ONE

We thank the editor (s) and reviewers for your careful reading of the manuscript and your constructive remarks. We feel we have fully addressed the concerns/comments raised by the reviewers and the editors and we thank you all for your insights

We have modified this manuscript according to the comments and suggestions made by the reviewers and editors, and we believe that the manuscript has significantly improved. Our detailed point-by-point response (in blue) to the comments raised by the editor (s) and reviewers is given below.

Moreover, the revised manuscript is marked in colored text in the manuscript file ('Revised Manuscript with Track Changes). All the suggestions and corrections given by the reviewers are incorporated in the revised manuscript.

We believe that our manuscript has been considerably improved and suitable, and hope that our revised manuscript “**Spatial regression models to assess variations of composite index for anthropometric failure across the administrative zones in Ethiopia with ID** PONE-D-21-35792” is acceptable for publication in the plos one journal.

On behalf of the co-authors, I would like to thank you once again for your consideration of our work and for inviting us to submit the revised manuscript.

We thank you for your consideration and look forward to your decision.

Yours sincerely,

Haile Mekonnen Fenta

Additional Editor Comments (if provided):

1. Abstract
(1) In the last sentence of 'Background', I think the statement of the aim is not accurate, which is inconsistent with the research content and conclusion. 'To determine the geographic distribution of CIAF and identify the influencing factors of it' might be more appropriate.

Our response: Thank you for your contractive comments and suggestions. We have read your comments carefully and made every effort to address them. It has to be corrected
(2) In the last line of 'Results': 'significant influencing factors' might be more preferred than 'covariates'.

Our response: Thank you, correction are made accordingly.

(3) In the second sentence of 'Conclusion': 'and improving children's wellbeing contribute to eradicating the CIAF of under nutrition' is not exact. That is not a proven fact in this study, but a conjecture based on research results.

Our response: Thank you for your comments and suggestions. It has been managed in the revised manuscript.

2. Introduction
(1) In the third sentence of the first paragraph, what kind of higher risk are children under 5 years old at? under nutrition or mortality?

Our response: Now it is corrected.

(2) I suggest that the content of the first paragraph needs to be appropriately streamlined, such as, the statements about relationship between under-nutrition and child mortality was mentioned in two different places; what do you mean by this sentence 'These studies employed classical models such as generalized linear (mixed) models.'

Our response: Thank you for your valuable suggestions, the amendment has been made accordingly in the revised manuscript.

(3) What are the practical benefits of studying malnutrition at the lower administrative level? 'This gives biased estimates since...', what does 'this' specifically mean? I would like to see more details about what problems have been unsolved in previous studies, and the importance of spatial analysis on this topic.

Our response: Thank you for your concern here. This study is conducted at the administrative zones in Ethiopia. This is because, in the country, the zonal health departments have the mandate to plan, follow up, monitor, and evaluate health activities of Woreda health offices and the diferent Woredas in the same Zone are relatively similar in many respects. Moreover, the administrative Zones are mainly ethnic-based, and the assessment of the Zones provides cultural practices regarding staple food and the geographic environment of the community in the Zones.

3. Methods and analysis
(1) I suggest that the contents in the second paragraph of 'Study area and data' should be moved to background.

Our response: Thank you, correction has been made accordingly.
(2) More details about the data used in this study (including CIAF and covariates) should be given, such as data source, sample size, the process of data collection, quality.

Our response: thank you for your comments and suggestions, really appreciated. Correction has been made according to your valuable comments.
(3) Please explain what standards of the age-specific norm are used? WHO standards or local standards?

Our response: The observed prevalence of the measures was categorized based on WHO’s classification for assessing severity of the situation for each anthropometric measure at the population level. This is incorporated in the revised manuscript.
(4) More clear definitions about covariates should be given, such as 'the proportion of children with vitamin A', what is the age range of children? What is the definition of vitamin A supplementation?

Our response: The age of children in this context means under-five and it is corrected.

(5) Are there any missing values in your dataset? What are the methods of dealing with missing data?

Our response: There were few missing values (less than 1%) and which were excluded from the analysis.
(6) I guess the data used in this study is panel data. The author described in detail how to deal with spatial factors, but did not elaborate on how to deal with temporal factors. When you modeling the relationship between CIAF and covariates, whether the zone level population has been corrected?

Our response: Thank you for your concern. The temporal effects were not included in this paper. This is clearly stated in the discussion section as “limitation and Strength” of the paper. Moreover, the population at the zone level has been corrected. This is directly obtained from the DHS survey datasets.
(7) What statistical tools are used in this study and what is the statistical significance level?

Our response: Thank you for your question. In this paper our aim is to explore the spatial variation of the outcome variable (CIAF) and the covariates among the administrative zones. Hence, we aggregated all the variables based on the zones and different spatial statistical models were used.

3. Results
(1) In the first paragraph, 'Twenty five percent of the 72 zones in Ethiopia had 48% of undernourished (CIAF) children' is not exact description. Please modified as ' Twenty five percent of the 72 zones in Ethiopia had less than 48% of undernourished (CIAF) children'.

Our response: Thank you for your comment; it had been corrected in the revised manuscript.
(2) The decimal places of the data in Table 2 shall be consistent. Keeping one decimal fraction for proportion is enough, I think.

Our response: Thank you, correction made accordingly
(3) All the statement about the statistical methods or the process of modeling in the results section should be moved to the methods section.

Our response: Yes some points are deleted, it is redundant and more elaboration were given in the methodology section.
(4) I would like to see the standard error of each parameter in each statistical model, please shown in Table 3.

Our response: Thank you so much, now it is corrected in the revised manuscript.
(5) Given that some independent variables may have serious collinearity, I'm very curious about how the author dealt with this problem.

Our response: Thank you so much for your concern, this is one of the PhD dissertation part so that the multicolliniearity issue was checked. Now in the revised manuscript the results are included and the result revealed that multicolinearity is not a big deal. .
(6) I would like to suggest that if the authors could give priority to the different important influencing factors that could be intervened for each region or zone, it will be more meaningful for policy makers.

Our response: yes now it has been managed and the points are included in the revised manuscript.
4. Discussion
I suggest the discussion needs to be much improved. What are the new findings of this study, and what are the differences from previous studies, especially the influencing factors of CIAF weighted by spatial factor.

Our response: Thanks, now it is improved.
5. Others. The language of this manuscript should be improved. The quality of figures needs to be much improved.

Our response: Yes managed

Reviewers' comments:

Reviewer's Responses to Questions

**Comments to the Author**

1. Is the manuscript technically sound, and do the data support the conclusions?

The manuscript must describe a technically sound piece of scientific research with data that supports the conclusions. Experiments must have been conducted rigorously, with appropriate controls, replication, and sample sizes. The conclusions must be drawn appropriately based on the data presented.

Reviewer #1: Yes

Reviewer #2: Partly

2. Has the statistical analysis been performed appropriately and rigorously?

Reviewer #1: Yes

Reviewer #2: Yes

3. Have the authors made all data underlying the findings in their manuscript fully available?

The [PLOS Data policy](http://www.plosone.org/static/policies.action#sharing) requires authors to make all data underlying the findings described in their manuscript fully available without restriction, with rare exception (please refer to the Data Availability Statement in the manuscript PDF file). The data should be provided as part of the manuscript or its supporting information, or deposited to a public repository. For example, in addition to summary statistics, the data points behind means, medians and variance measures should be available. If there are restrictions on publicly sharing data—e.g. participant privacy or use of data from a third party—those must be specified.

Reviewer #1: Yes

Reviewer #2: Yes

4. Is the manuscript presented in an intelligible fashion and written in standard English?

PLOS ONE does not copyedit accepted manuscripts, so the language in submitted articles must be clear, correct, and unambiguous. Any typographical or grammatical errors should be corrected at revision, so please note any specific errors here.

Reviewer #1: Yes

Reviewer #2: No

5. Review Comments to the Author

Please use the space provided to explain your answers to the questions above. You may also include additional comments for the author, including concerns about dual publication, research ethics, or publication ethics. (Please upload your review as an attachment if it exceeds 20,000 characters)

Reviewer #1: Review Report
This paper aims to evaluate the existence of spatial dependence on the outcome of undernutrition and the respective covariates in Ethiopia across the administrative zones. Specifically, to:
determine and explore the overall and local spatial dimension of CIAF among the under-five children in Ethiopia, (2) identify the factors and their effects for the spatial disparities in the average CIAF among the under-five children (3) compare the spatial model's performance to the standardized regression model, and (4) address the spatial vulnerability of the community to child undernutrition at the zonal level using the EDHS dataset.. The manuscript is written in a structured style, and is easy to follow. However, the language should be checked, and some aspects of the paper need extra work or clarification.
Please find the following specific comments:

Methods/Methodology
1-Although the authors drew the models in figure 3, the explanation of how the models evolve is not very clear. A clearer explanation of the models would be helpful in understanding the results from each.

Our response: Thank you for your valuable comments. Your suggestion helped us to improve the manuscript once again.
2-In table 1, the authors mentioned that (CIAF) is binary variable and later the number of CIAF cases observed in the zone i (i=1, 2, . . . , 72), denoted by , was assumed to follow a Poisson distribution. Please, provide a clear explanation about that approximation.

Our response: Thank you for your question. Table 1 is about weather a child is undernourished or not and then if a child is undernourished it will be counted. So in this manuscript we count the number of under-five children for each of the second administrative zones.
3-The authors mentioned that it is spatial data, what are the sample sizes of these variables in each region.

Our response: This is a very good point. Now this is elaborated in the methodology section.
4- The notations seems not consistent through the paper. For example, SAC: spatial autocorrelation model and SAR: spatial autoregressive model. GNS: general nesting spatial model and in table 3, what is GNNS?

Our response: Thanks, not corrected in the revised format.
5-In the methodology, the authors mentioned that “The spatial effects model is summarized in Fig.3”. In that figure, OLS is mentioned. Is it a spatial effects model? It will be “The spatial effects models as well as the OLS model……”

Our response: This is a critical comment. Corrected in the revised manuscript.

6-Which software was used for the analysis?

Our response: We used R statistical software and the statement is added in the revised manuscript.
7-Various methods can be used to estimate spatial models, which one was used in this article?
Our response: We have created the 72 by 72 weighted matrix and we developed our own R codes to do the analysis as provided in the methodology section. Hence the maximum likelihood estimation was used.
Abstract
1- Line 2 “……….but the spatial heterogeneity in these relationships across the administrative zones remains unstudied”. I find that this statement should be rewritten since according to Fenta et al. (2021). Research entitled “Spatial data analysis of malnutrition among children under five years in Ethiopia”, spatial heterogeneity was used in the models.

Our response: Thank you for your critical view. That paper was from same authors and in that paper we considered the spatial effects on the outcome variable only. Some modification has been made in the revised manuscript.
2- Line 4 “……zones autocorrelation structure in the under-five children’s undernutrition studies. This should be rewritten.

Our response: Thank you so much, it has been managed and corrected in the revised manuscript.
3- Line 6. “We obtained the zonal-level …….. data for the under-five children in Ethiopia from the Ethiopian Demographic and Health Survey (EDHS) dataset”. It will be “We used the zonal-level …. data for the under-five children in Ethiopia from the Ethiopian Demographic and Health Survey (EDHS) dataset”.

Our response: Thank you, it has corrected in the revised manuscript.
4- The spatial autocorrelation model (SAC) was the best fit based on the AIC and BIC criteria. I am not able to identify the BIC values in the tables. I don’t think if those values were generated.

Our response: Thank you for your concern. The lowest AIC is the best model and same is true for BIC. Unfortunately there is no built in functions in spatial regression models for BIC.
5- The Confidence intervals are not shown in table 3.

Our response: This is a critical point. Now the standard deviation is added and due to the space, the confidence interval is missed. In fact, the confidence interval can be computed from the parameter estimate and the standard errors of its estimate.
6- Is mothers’ literacy rate the same with % women with illiteracy in table 3? If yes, use the same name. Also, the value in the table and which described are not the same. 0.605 vs 0.065

Our response: Thank you so much. The value is corrected now, it was wrongly put. The prevalence and rate is simply proportion in our case and more explanation is given in Table 1 (methodology section).
7- Is minimum temperature the same with mint? The value described is not correct -0.158 vs -0.133. What about the p value?

Our response: Yes it is. The variable description is given in Table 1. The p-value definition is provided under Table 3.
8- ….” The significant covariates may provide insights for policymakers for considerations.” It should be rewritten.

Our response: Thank you so much, now it is rewritten in terms of significant variables.
Results
1-It would be helpful to include a map of Ethiopia with the administrative divisions shown the generated latent spatial effects with a Moran’s I.

Our response: Thank you, this is very important point. However, our intention in this manuscript was to assess the spatial distributions of both the outcome and independent variables.

2- Note of the table 2, …. “*, ** and ***= Moran’s I are significant at 10, 5, and 1% level”. It will be ““*, ** and ***= Moran’s I are significant at 10%, 5%, and 1% level, respectively”.

Our response: Managed in the revised manuscript.
3- What is N in table 3?

Our response: Thank you. N is the number of administrative zones in Ethiopia. It has been taken from the shapefiles.
4- Use the same number of decimal. For example, “…. in Ethiopia was 52%, with the mothers’ illiteracy rate of 73%, and with 38.44% …”.

Our response: managed in the revised manuscript
Discussion
What is U5C?

Our response: Thank you for your critical view. It stands for under 5 children in the study. In it is corrected in the revised manuscript.

The results are not well discussed.

Our response: Thank you so much for your concern. Now the correction is included in the revised form.
Decision
The paper can be published upon addressing the above comments.

Our response:

Reviewer #2: Spatial regression models to assess variations of the composite index for anthropometric failure across the administrative zones in Ethiopia

Summary: Overall, the manuscript is good for statistical analysis or method paper. The authors were trying to assess the variation of CIAF across the different Zones of Ethiopia. Using a spatial regression model is a new technique to assess the CIAF variation. But it is unclear how this study could help the policy makers/ clinicians/ others to take necessary measures to reduce malnutrition. The public health background is absent for this paper, as CIAF is for the policymakers but not for clinicians. Here are some comments from the authors which would help the paper to be in better shape for the readers and policymakers.

Our response: Thank you for your appreciations. One of the aims of this study is to assess the spatial correlations of undernutrition among the administrative zones. Detecting the problem of undernutrition and its variations among different administrative zones provides deeper insight into the country's health priorities for under-five children and for zonal health departments to plan, follow up, monitor, and evaluate health activities at lower levels. Since there are cultural and climatic variations among the administrative zones, resulting in different practices regarding staple food in the zones, it would be important to assess the CIAF at zone level. Hence, the limited resources will be allocated to those disadvantage zones. This is the contribution of the manuscript.

Abstract: I am a bit confused, about how the authors write the conclusion based on the results shown in the abstract. “Results from the SAC model suggested that the mothers’ literacy rate (0.065, pvalue0.001) ….” What is 0.065? mentioning this would be easy for a reader to understand the values. The background section needs to write the importance of this study, the authors mainly mentioned the gap! Is there any other public health implication for conducting this study? Have any role to prevent malnutrition?

Our response: Thank you for your valuable comments and suggestions. The abstract is amended accordingly.
The keyword should contain CIAF.

Our response: Thanks now incorporated in the revised manuscript.
Introduction: In the 1st paragraph, the last two lines need references. The aim of the study is, it is a kind of method paper. Better to have some public health impact in the introduction section. How This can help to reduce malnutrition/ policymakers to understand the result of this study for taking mitigation strategy for tackling malnutrition.

Our response: Thank you. Amendment has been made and the comments are incorporated in the revised version.
Study area: Need references along with the figure. Why only 2000-2016 data were used? Not the recent one? Up to 2020 or more?

Our response: This is a very good point. The references are added. The country (Ethiopia) has four standard DHS datasets (2000, 2005, 2011 and 2016) and in this study we include only the standard DHS datasets. The recent 2019 is the min DHS which is not representative.

Variables of the study: How Z scores were calculated? Better to mention in this section, as CIAF is the important one in this paper. For readers who are from a non-statistical background, I think the table is not sufficient, the author can elaborate on the covariates; how they were collected, how they were categorized, and how defined.

Our response: Thank you for your suggestions. The points you had raised are incorporated in the revised manuscript.
Methods: Authors wrote the methods section in detail, which is highly appreciated.

Our response: Thank you for your appreciation. In fact the aim of this paper was also contributing this new methodology for health sciences.
Result: It is clear cut and shown in a good manner fashion.

Our response: Thank you.

Discussion: After reading the discussion section, it seems incomplete to me. It’s ok that no other research did not use this type of data, but authors can compare their findings with the studies outside Ethiopia, or other studies which are globally acceptable for similar types of research.

Our response: Yes, now the discussion part is revised.
“In the SAC model, which is considered to be the best, the signs of below minimum dietary diversity score, illiteracy rate, the proportion of low BMI index, and precipitation variables were positive and significantly different from zero.” -are those findings very common and why these were observed among Ethiopia? It seems the result section is a bit more elaborated in the discussion section. The discussion needs to be rewritten.

Our response: The result is checked and the description is corrected.
Is there any strength or limitation of this analysis/ study? Which might be mentioned at the end of the discussion section.

Our response: Thank you so much. The limitations and strength of this paper is incorporated in the discussion section as per your recommendation.
Conclusion: “The spatial models allow the development of models that will significantly assist decision-makers in the improvement of undernutrition.”- I have no idea how? It is not clear in the conclusion/discussion section. Policymakers/ decision makers are not super specialized with statistical methods always. For the policy implication, a method paper is not a good option according to my knowledge. The recommendation should be changed or rewritten.

Our response: Yes now corrected.
Minor comments: Language can be better, sometimes it seems sentences are alone, not meaningful. There were some issues with spacing and font.]

Our response: Thank you, language has been checked by professionals.
